# Increased risk of second cancers at sites associated with HPV after a prior HPV-associated malignancy, a systematic review and meta-analysis

**DOI:** 10.1038/s41416-018-0273-9

**Published:** 2018-11-28

**Authors:** Duncan C. Gilbert, Katie Wakeham, Ruth E. Langley, Claire L. Vale

**Affiliations:** 1 MRC Clinical Trials Unit at UCL, Institute of Clinical Trials and Methodology, 90 High Holborn, London, UK; 2 Sussex Cancer Centre, Royal Sussex County Hospital, Eastern Road, Brighton, UK

**Keywords:** Cancer epidemiology, Cancer epidemiology, Tumour virus infections

## Abstract

**Background:**

High-risk human papilloma viruses (HPV) are a causative agent of anogenital and oropharyngeal cancers. Patients treated for a preinvasive or invasive HPV-associated cancer may be at increased risk of a second such malignancy.

**Methods:**

We performed a systematic review and random effects meta-analysis to estimate the risk of HPV-associated cancer after prior diagnosis. Studies reporting second cancers at anogenital and oropharyngeal sites after prior diagnoses (preinvasive/invasive HPV-associated cancer) were identified. Studies reporting standardised incidence ratios (SIRs) were included in formal meta-analyses of second cancer risk. (PROSPERO ID: CRD42016046974).

**Results:**

Searches returned 5599 titles, including 60 unique, eligible studies. Thirty-two (98 comparisons) presented SIRs for second cervical, anal, vulvo-vaginal, penile, and/or oropharyngeal cancers, included in the meta-analyses. All studies (and 95/98 comparisons) reported increased cancers in the population with previous HPV-associated cancer when compared to controls. Pooled SIRs for second primary cancers ranged from 1.75 (95% CI 0.66−4.67) for cervical cancer after primary anal cancer, to 13.69 (95% CI 8.56−21.89) for anal cancer after primary vulvo-vaginal cancer.

**Conclusions:**

We have quantified the increased risk of second HPV-associated cancer following diagnosis and treatment for initial cancer or preinvasive disease. This has important implications for follow-up, screening, and future therapeutic trials.

## Background

High-risk human papilloma viruses (HPV) are acknowledged as causing cancers of the cervix, anus, vulva, vagina, penis and oropharynx. The incidence of HPV-associated anogenital and oropharyngeal cancers is rising in the developed world and is a major cause of morbidity and mortality across low and middle-income countries. Approximately 5% of all cancers worldwide are caused by HPV^1^ with the proportion of cancers attributable to HPV at each site ranging from 50% (vulval) to ~90% (anal).^2^ Cancers arising at these sites have marked biological similarities^3^ and treatment protocols. Many HPV-associated cancers and precancerous lesions (termed intraepithelial neoplasia) present with early disease and cure rates following surgical excision (i.e. for early-stage cervical cancers or anal intraepithelial neoplasia) are excellent.^4,5^ For patients with locally advanced disease (for example head and neck or anal squamous cell carcinomas) treatment typically involves radical chemo-radiotherapy, with relatively high rates of long-term survival.^6,7^

Although patients diagnosed with primary HPV-associated cancers then are often cured, they remain at risk of second HPV-associated malignancies. A number of factors likely contribute to this increased risk including prior exposure to high-risk subtypes of HPV where sexual behaviour promulgates this risk.^8^ Intra-patient transmission of HPV across the various anatomical sub sites of the anogenital regions is recognised. Additionally, there is evidence to support underlying biological susceptibility to HPV-associated cancers where candidate gene approaches or genome-wide association studies suggest that polymorphisms within immune pathways might play a role. Variants of the TGF beta receptor 1 have been associated with HPV-associated head and neck cancer ^9^ and MHC variants linked with cervical cancer.^10^ A number of registry and other studies report incidence rates of second primary HPV-associated cancer, typically focussing on a single primary tumour and a subset of the potential second cancers. However, a more accurate estimate of this risk is required following treatment of the initial cancer to understand the need for and inform the design of follow-up and surveillance protocols. It would also facilitate the investigation of additional treatments in the future such as novel screening or therapeutic vaccination strategies to reduce the risk of second cancers.

We therefore conducted a systematic literature review and meta-analysis to estimate the overall rates of second HPV-associated cancers following treatment of an initial such tumour.

## Methods

A protocol, including the full methods for this review, is available from http://www.crd.york.ac.uk/PROSPERO/display_record.asp?ID=CRD42016046974.

### Study eligibility

#### Systematic review

To be comprehensive, studies were considered eligible for inclusion in the systematic review if they reported second HPV-associated cancers after an initial index cancer (or preinvasive, in situ neoplasia) at a site associated with HPV infection, i.e. invasive cervical, vaginal, vulval, anal, or penile cancers or their associated preinvasive lesions (CIN/VAIN/VIN/AIN/PIN) or cancers of the oropharynx (tonsil and tongue base). These included previous systematic reviews, cohort studies including from cancer registries, and phase III trials of radical treatment that report second cancers. All eligible studies were included in the results of the systematic review.

#### Meta-analysis

To limit ascertainment bias, only studies that measured and reported the same statistics using the same measures were included in the formal meta-analysis. Studies reporting the risk of second cancers in a population affected by the primary index cancer compared with the risk of those cancers in a contemporary control population not affected by the primary cancer (e.g. derived from SEER data) were eligible for inclusion in the meta-analysis. Specifically, this must have been presented as a standardised incidence ratio (SIR), calculated by dividing the observed incidence of second primary malignancies (SPM) by the incidence for the general population, measured from the rest of the registry unaffected by the primary cancer in question.^11^

### Study identification

To identify eligible studies that reported subsequent incidences of cancers including, but not limited to, those known to be associated with HPV after an initial diagnosis, we developed a comprehensive search strategy for MEDLINE. The search strategy included MeSH and free-text terms for each of the HPV-associated cancer sites or precancerous in situ disease states, namely cervix, vagina, vulva, oropharynx, penis and anus, as well as for each of the relevant study types and for second primary cancer. The strategy used is given in Appendix 1 (supplementary material). Web of Science, ASCO, ESMO/ECCO databases and conference proceedings of the International Papillomavirus Society (IPVS) were also searched for relevant articles or abstracts. Reference lists of included articles were manually screened to retrieve any additional eligible studies. Searches were updated until 7 July 2016.

### Data extraction

Data were extracted from the reports of all studies identified as being eligible for inclusion in the systematic review using a predefined form, including where available: origin of patient population (registry, single centre cohort, randomised trial cohort); time points of initial diagnosis; number at risk; subsequent incidence of HPV-associated cancers and precancerous in situ disease of the anogenital region (cervical, vulval, vaginal, penile, anal) and the oropharynx (specifically, base of tongue and tonsil). In addition, for studies to be included in the formal meta-analysis, SIR and associated statistics for each second primary HPV-associated cancer were also extracted.

### Risk of bias/quality assessment of studies

Since all eligible studies were of cohort design, the Newcastle–Ottawa quality assessment scale^12^ was used to evaluate methodological quality. A meta-analysis of observational studies in epidemiology (MOOSE) checklist ^13^ was completed and is included in the Supplementary Materials.

### Statistical analysis

Absolute numbers of second cancers and associated standardised incidence rates (SIR)^11^ were tabulated from each study, organised according to the site of index primaries. Where SIR for relevant individual sites of second primary cancer (vulval and vaginal cancers or tonsil and tongue base) were reported separately, data were pooled using a random effect meta-analysis to obtain a single SIR for the combined site (i.e. vulvo-vaginal and oropharyngeal).

For each second cancer type (cervix, anal, oropharynx, penile and vulvo-vaginal) the SIRs and associated statistics from the individual studies were combined in a formal meta-analysis according to the index cancer site, to obtain an estimate of the risk of independent second primary cancer following individual index primaries. Chi-square tests for interaction were used to investigate whether there were any substantial differences in the risk of second primary cancers between groups of studies based on primary cancer type. SIRs and associated statistics for second primary cancer at the same location as the index HPV-associated cancer were considered separately.

Statistical heterogeneity and inconsistency^14^ were also assessed within the subgroups of studies based on the index HPV-associated cancer for each second primary cancer type. To account for expected heterogeneity between studies, a random effects meta-analysis model was used.^15^ Analyses were conducted using the IPDmetan command^16^ in Stata version 14.

## Results

### Eligible studies

**Fig. 1:**
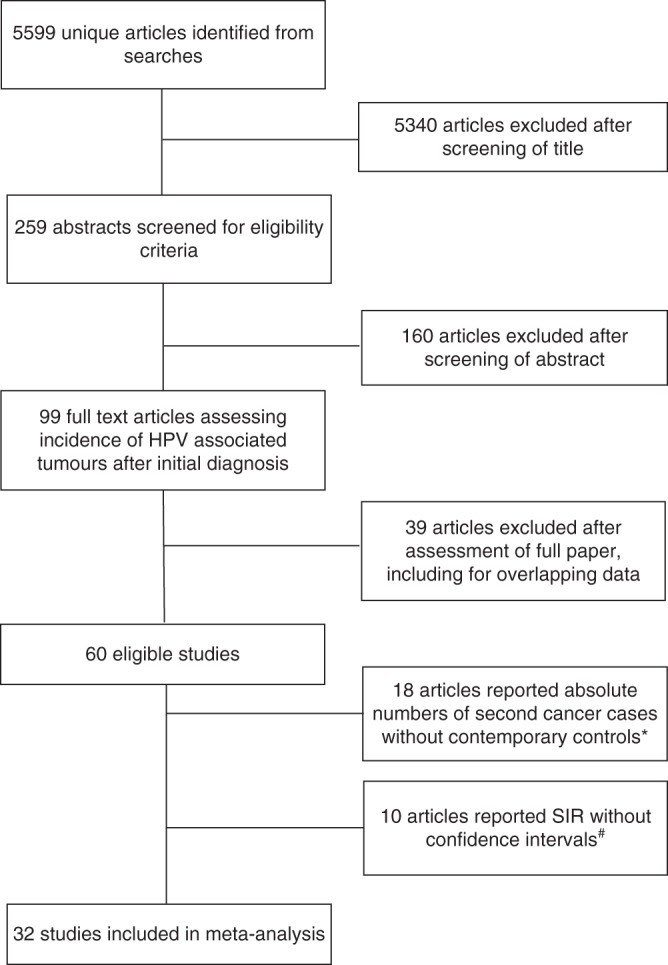
PRISMA flow diagram of identification and selection of eligible studies. *Included in discussion with respect to estimation of absolute risk of subsequent HPV-associated cancer, Table S1. ^#^Included in Table S2

Searches returned 5599 titles, which were screened for eligibility (Fig. 1). Sixty studies fulfilled the criteria for the systematic review; however, 18 studies^17–32^ reported institutional cohorts with absolute numbers of second primaries (Table S1) and a further 10 studies^33–42^ reported second primaries and SIRs without providing either confidence intervals or standard errors (Table S2), so they could not be included in the formal meta-analyses. The remaining 32 studies^43–74^ from large institutional, regional or national cancer registries (representing 16 countries), all reported SIRs and associated statistics and are therefore included in the meta-analysis (two pairs of studies reported overlapping data from the same sources and were combined). These 32 studies comprised 3,759,726 patients and yielded 98 comparisons of individual sites of HPV-associated cancer after an index case. Characteristics of the 32 studies are shown in Table 1. All 32 studies were assessed as having reasonable quality (score range: 5−8) according to the Newcastle Ottawa framework. A MOOSE checklist^13^ is included in the supplementary materials.

**Table 1:** Studies reporting SIR for second primary HPV cancers after an initial HPV-associated tumour

Author^ref.^	Study type	Country and data source	Study population, definition and inclusion criteria	Primary cancer diagnosis timeframe and follow-up duration	*N*	Second primary cancer type(s); number of cases	SIR (95% CI)	Notes
First primary cancer: Cervical/CIN								
Bjorge et al.^43^	Retrospective cohort study	Norway; Cancer Registry of Norway	Women with diagnosis of cervical carcinoma in situ; second cancer diagnosis ≥ 1 year following CIS diagnosis	1970−1992; 9.1 years (mean)	37,001	Cervix; 11 Oropharynx; 9 Vulvo-vaginal; 32	1.26 (0.6−2.3) 2.88 (2.43−3.42) 4.04 (2.76−5.70)	Incidence of second primary tongue and tonsil reported separately and pooled for this analysis
Chaturvedi (same data set)^44, 45^	Retrospective cohort study	Denmark, Finland, Norway, Sweden, USA; data from 13 population-based cancer registries	One-year survivors of cervical cancer	1943–2001; 12.2 years (mean)	104,760	Vulvo-vaginal; 497 Anal; 817 Oropharynx; 84	4.81 (4.40−5.25) 1.84 (1.72−1.98) 1.49 (0.97−2.29)	Incidence of second primary tongue and pharynx reported separately and pooled for this analysis
Chen^46^	Retrospective cohort study	Taiwan; Taiwan Cancer Registry	Women with initial diagnosis of cervical cancer and complete data available	1979−2008; 8.18 years (mean)	52972	Vulvo-vaginal; 137 Oropharynx; 37	10.48 (8.80−12.39) 1.18 (0.83−1.62)	
Edgren and Sparen^47^	Retrospective cohort study	Sweden; Swedish Cancer Register	Women with diagnosis of CIN Grade 3 diagnosis; second cancer diagnosis ≥ 1 year following CIN diagnosis	1968−2004; 27 years (median)	2,302,024	Vulvo-vaginal; 173 Anal; 131	3.74 (1.55−9.04) 2.81 (1.29−5.44)	Incidence of second primary vulva and vaginal cancers reported separately and pooled for this analysis
Evans et al.^48^	Retrospective cohort study	UK; Thames Cancer Registry	Women with a diagnosis of CIN 3 Women with a diagnosis of cervical cancer	1960−1999; 8.0 years (mean) 1960−1999; 6.7 years (mean)	59,579 21,605	Cervix; 194 Vulvo-vaginal; 61 Anal; 23 Oropharynx; 20 Cervix; 3 Vulvo-vagina; 26 Anal; 18 Oropharynx; 16	2.8 (2.4-3.2) 9.08 (2.22−37.09) 5.9 (3.7−8.8) 1.2 (0.8−1.9) 0.1 (0−0.3) 3.91 (0.96−16.01) 6.3 (3.7−10.0) 1.4 (0.8−2.2)	Study reports second cancer incidences according to whether the primary event was CIN 3 or invasive cancer. Incidence of second primary vulva and vaginal cancers reported separately and pooled for this analysis
Fisher et al.^49^	Retrospective cohort study	USA; Michigan Cancer Surveillance Records and US census data	Women living in Michigan, diagnosed and registered with cancers of the lower anogenital tract	1985–1992; 8 years (maximum)	1565	Cervical; 5 Vulvo-vaginal; 6	3.6 (1.2−8.3) 44.3 (16.2−96.5)	
Gaudet et al.^50^	Retrospective cohort study	Canada; British Columbia Cancer Agency cervical cancer screening programme database and British Columbia Cancer Registry	Women with pathological diagnoses of CIN 2 or 3	1985−2005; 10.1 years (median)	54,320	Vulvo-vaginal; 143 Anal; 20	4.20 (1.87−9.43) 1.75 (0.43−4.65)	Incidence of second primary vulva and vaginal cancers reported separately and pooled for this analysis
Hemminki et al.^51^	Retrospective cohort study	Sweden; Swedish Family Cancer Database and Swedish Cancer Registry	Women with diagnoses of primary invasive cervical cancer	1958−1996; Average follow-up unknown	17,234	Cervix; 46 Genital; 67 Anal; 16 Oropharynx; 33	0.84 (0.62−1.10) 5.91 (4.58−7.41) 4.22 (2.41−6.55) 2.20 (1.51−3.01)	
Hemminki et al.^52^	Retrospective cohort study	Sweden; Swedish Family Cancer Database and Swedish Cancer Registry	Women with diagnoses of CIS	1958−1996; Average follow-up unknown	117,830	Cervix; 758 Genital; 155 Anal; 68 Oropharynx; 79	2.30 (2.14−2.47) 3.68 (3.12−4.28) 3.75 (2.91−4.69) 1.69 (1.33–2.08)	
Jakobsson et al.^53^	Retrospective cohort study	Finland; Finnish National Hospital Discharge Register and Finnish Cancer Registry	Women receiving surgical treatment for CIN	1986−2004; 8.4 years (mean)	26,876	Cervix; 23 Vulvo-vaginal; 17: Anal; 3	1.69 (1.07–2.53) 6.84 (4.08−11.48) 3.56 (0.73−10.4)	Incidence of second primary vulva and vaginal cancers reported separately and pooled for this analysis
Kalliala et al.^54^	Retrospective cohort study	Finland; Finnish Population Registry and Finnish Cancer Registry	Women receiving surgical treatment for CIN at Helsinki Central University Hospital	1974−2001; 11.9 years (mean)	7564	Cervix; 22 Vulvo-vaginal; 11 Anal; 3	2.8 (1.7−4.2) 6.86 (2.40−19.65) 5.7 (1.2−17.0)	Incidence of second primary vulva and vaginal cancers reported separately and pooled for this analysis
Levi et al.^55^	Retrospective cohort study	Switzerland; Swiss Cancer Registry of Vaud	Women with diagnoses of CIS	1974−1993 10.1 years (average)	2190	Cervix; 10	3.4 (1.6−6.3)	
Lim et al.^56^	Retrospective cohort study	South Korea; Korea Central Cancer Registry	Women diagnosed with cervical cancer	1993−2010; 7.34 years (mean)	72,805	Vulvo-vaginal; 24 Anal; 11 Oropharynx; 9	4.98 (1.41−17.61) 2.42 (1.21−4.32) 1.33 (0.63–2.78)	Incidence of second primary vulva and vaginal cancers and tongue and tonsillar cancers reported separately and pooled for this analysis
Mitchell et al.^57^	Retrospective cohort study	Australia; Victorian Cytology Gynaecological Service records	Women with a histologically confirmed diagnosis of CIN	1974−1976;	1281	Cervix; 6	19.8 (2.4−163.5)	
Neumann et al.^58^	Retrospective cohort study	France; K2 database	Men and women with potentially HPV-related primary cancer diagnoses	1989−2004; 3.1 years (median)	6049 women	Vulvo-vaginal; 8 Anal; 5	11.74 (5.23−25.99) 5.42 (1.75−12.64)	Incidence of second primary vulva and vaginal cancers reported separately and pooled for this analysis
Rabkin et al.^59^	Retrospective cohort study	USA; Connecticut Tumor Registry and National Cancer Institute SEER database	Women with first primary cervical cancer	1935−1988 /1973−1988; 8.5 years	9325	Vulvo-vaginal; 54 Anal; 12 Oropharynx; 47	5.6 (4.2−7.4) 4.6 (2.4−8.1) 2.2 (1.6–2.9)	Connecticut registry 1935−1988; Other US registries 1973−1988
Rose Ragin and Taioli^60^	Retrospective cohort study	USA; National Cancer Institute SEER database	Women with first primary cervical cancer	1973−2002; Average follow-up not reported	2618	Vulvo-vaginal: number of cases not reported: Anal: number of cases not reported: Oropharynx; 12	9.37 (2.96−29.79) 2.9 (1.7−4.5) 2.7 (1.4–4.7)	Incidence of second primary vulva and vaginal cancers reported separately and pooled for this analysis
Saleem et al.^61^	Retrospective cohort study	USA; National Cancer Institute SEER database	Women with a confirmed diagnosis of CIN; > 15 years old	1973–2007; 15.7 years (mean)	124075	Anal; 137	16.4 (13.7-19.2)	
Saleem et al.^61^	Retrospective cohort study	USA; National Cancer Institute SEER database	Women with a confirmed diagnosis primary cervical cancer; >15 years old	1973−2007; 11.4 years (mean)	43,669	Anal; 28	6.2 (4.1−8.7)	
Sand et al.^62^	Retrospective cohort study	Denmark; Danish civil Registration system and Danish Cancer Registry	Women born between 1918 and 1990, resident in Denmark between 1978 and 2012 with histological confirmation of CIN2 or CIN3	1978−2012; 11.5 years (mean) 1978−2012; 14.7 years (mean)	52,135 (CIN2) 104,155 (CIN3)	Vulvo-vaginal; 34 Anal; 32 Vulvo-vaginal; 168 Anal; 125	4.41 (1.39−13.94) 2.9 (2.0−4.1) 8.24 (1.99−34.22) 4.2 (3.5−4.0)	Incidence of second primary vulva and vaginal cancers reported separately and pooled for this analysis
Strander et al.^63^	Retrospective cohort study	Sweden; Swedish Cancer Registry	Women diagnosed and treated for CIN3	1958−2002 Average follow-up not reported	132,493	Cervical; 881 Vaginal; 111	2.34 (2.18−2.50) 6.82 (5.61−8.21)	
Svahn et al.^64^	Retrospective cohort study	Denmark; Danish Cancer Registry and danis Pathology Databank	Women born between 1918 and 1990, living in Denmark 1995−2012 and diagnosed with CIN3	1995−2012 Average follow-up not reported	101,974	Oropharyngeal; 47	2.51 (1.86−3.39)	
First primary cancer: Vulvo-vaginal/VIN								
Hemminki et al.^51^	Retrospective cohort study	Sweden; Swedish Family Cancer Database and Swedish Cancer Registry	Women with diagnoses of primary invasive genital cancer	1958−1996; Average follow-up unknown	2528	Cervical; 7 Genital ; 15 Anal; 6 Oropharynx; 9	1.88 (0.75−3.54) 8.81 (4.92−13.84) 13.97 (5.03−27.39) 4.65 (2.11−8.19)	
Neumann et al.^58^	Retrospective cohort study	France; K2 database	Men and women with potentially HPV-related primary cancer diagnoses	1989−2004; 3.1 years (median)	6049 women	*Vaginal primary*: Cervical; 2 *Vulvar primary*: Cervical; 3 Vaginal; 1 Anal; 1	13.70 (1.54–49.45) 12.10 (2.43−35.36) 25.84 (0.34−143.95) 11.77 (0.15−65.51)	Reported separately for primary vulvar and vaginal cancers
Saleem et al.^61^	Retrospective cohort study	USA; National Cancer Institute SEER database	Women with a confirmed diagnosis of VIN; >15 years old	1973−2007; 8.9 years (mean)	6792	Anal; 55	22.2 (16.7−28.4)	
Saleem et al.^61^	Retrospective cohort study	USA; National Cancer Institute SEER database	Women with a confirmed diagnosis of invasive vulvar cancer; >15 years old	1973–2007; 7.1 years (mean)	9950	Anal; 28	17.4 (11.5−24.4)	
Saleem et al.^61^	Retrospective cohort study	USA; National Cancer Institute SEER database	Women with a confirmed diagnosis of Vaginal in situ; >15 years old	1973−2007; 11 years (mean)	1463	Anal; 5	7.6 (2.4−15.6)	
Saleem et al.^61^	Retrospective cohort study	USA; National Cancer Institute SEER database	Women with a confirmed diagnosis of invasive vaginal cancer; >15 years old	1973−2007; 4.5 years (mean)	3257	Anal; 25	1.8 (0.2−5.3)	
First primary cancer: Anal/AIN								
Frisch et al.^65^	Matched case-control study using	Denmark; Danish Cancer Registry (cases) and Central population register (controls)	Patients with diagnoses of primary invasive epidermoid anal cancer	1943−1989; Average follow-up (men): 5.1 years Average follow-up (women); 5.6 years	955	Cervical; 2 Vulvo-vaginal; 5 Penile; 7	1.6 (0.1−4.5) 12.3 (4.0−28.7) 1.8 (0.7−3.7)	
Hemminki et al.^51^	Retrospective cohort study	Sweden; Swedish Family Cancer Database and Swedish Cancer Registry	Men and women with diagnoses of primary invasive anal cancer;	1958−1996; Average follow-up unknown	334 men 744 women	Genital; 2 Oropharyngeal; 2 Cervical; 1 Genital; 2 Anal; 1 Oropharyngeal; 3	60.24 (5.68–172.66) 6.78 (0.64–19.42) 1.12 (0.00–4.39) 4.73 (0.45−13.55) 7.07 (0.00−27.71) 6.01 (1.13−14.75)	
Neumann et al.^58^	Retrospective cohort study	France; K2 database	Men and women with potentially HPV-related primary cancer diagnoses	1989−2004; 3.1 years (median)	6049 women	Cervical; 2 Oropharyngeal; 2	2.95 (0.3–10.66) 19.28 (2.17−69.60)	
Rabkin et al.^59^	Retrospective cohort study	USA; Connecticut Tumor Registry and National Cancer Institute SEER database	Men and women with first primary anal cancer	1935−1988/1973−1988; 5.12 years	530	Cervical; 2 Vulvo-vaginal; 2 Oropharynx; 4	11.3 (0.2−4.5) 2.5 (0.3−9.4) 1.0 (0.3–2.6)	Connecticut registry 1935−1988; Other US registries 1973−1988
Shah and Budhathoki^66^	Retrospective cohort study	USA; National Cancer Institute SEER database	Patients with a primary diagnosis of anal carcinoma	1992−2013; 87 months (median)	7661 (Men: 3196; Women: 4465)	Vulvo-vaginal; 24 Anal; 56 Penile; 1	10.154 (6.61−15.60) 30.87 (23.32−40.09) 2.93 (0.07−16.33)	Incidence of second primary vulva and vaginal cancers reported separately and pooled for this analysis
Sikora et al.^67^	Retrospective cohort study	USA; National Cancer Institute SEER database	Men with primary anal cancer diagnoses; aged 20 years or greater	1973−2004 5.3 years median	2080	Oropharyngeal; 10	5.99 (2.98–12.05)	Incidence of second primary tongue and tonsil reported separately and pooled for this analysis
First primary cancer: Penile/PIN								
Hemminki et al.^51^	Retrospective cohort study	Sweden; Swedish Family Cancer Database and Swedish Cancer Registry	Men with diagnoses of primary invasive genital cancer	1958−1996; Average follow-up unknown	1127	Genital; 3 Oropharyngeal; 2	12.71 (2.40–31.15) 2.57 (0.81–5.32)	
Sikora et al.^67^	Retrospective cohort study	USA; National Cancer Institute SEER database	Men with primary penile cancer diagnoses;	1973−2004; 6.7 years median	2217	Oropharyngeal; 12	4.74 (2.54–8.85)	Incidence of second primary tongue and tonsil reported separately and pooled for this analysis
First primary cancer: Head and neck (oropharynx)								
Bhattacharyya^68^	Retrospective cohort study	USA; National Cancer Institute SEER database	Cases from the SEER programme with primary head and neck cancer	1988−1999; Follow-up: 42.2 months (mean) Minimum follow-up at least 3 m	4122	Oropharyngeal: Number of cases not reported	5.951 (3.611−9.808)	Number of cases of second primary cancer not reported
Bosetti^69^ Chuang^70^ (same data set)	Retrospective cohort study	Australia, Canada, Denmark, Finland, Norway, Scotland, Singapore, Slovenia, Sweden, Spain; data from 13 population-based cancer registries	Cases with primary head and neck cancer diagnoses	1943−2000; 4.9 years (mean)	99,257	Oropharyngeal; 760	13.67 (10.06−18.58)	Incidence of second primary tongue and pharynx reported separately and pooled for this analysis
†Hemminki et al.^51^	Retrospective cohort study	Sweden; Swedish Family Cancer Database and Swedish Cancer Registry	Men and women with diagnoses of primary invasive oral cancers	1958−1996; Average follow-up unknown	10,780 (men) 3366 (women)	Anal; 2 Genital; 7 Oropharyngeal; 194 Cervical; 8 Vulvo-vaginal; 7 Anal; 1 Oropharyngeal; 71	2.68 (0.25−7.69) 3.7 (1.47−6.96) 10.16 (8.78–11.64) 1.73 (0.74–3.13) 3.74 (1.48−7.02) 1.88 (0.00−7.38) 29.43 (22.98−36.68)	
Jain et al.^71^	Retrospective cohort study	USA; National Cancer Institute SEER database	Men and women with diagnoses of primary invasive squamous cell carcinoma of the head and neck	1979−2008 Average follow-up not reported	16,877	Oropharyngeal; number of cases not reported	136.7 (107.1−171.8)	
Levi et al.^72^	Retrospective cohort study	Switzerland; Vaud and Neuchatel Cancer Registries	Men and women with diagnoses of primary oropharynx cancers	1974−2003; 3.9 years (average)	3092	Oropharyngeal; 233	31.7 (27.7−36.0)	
Morris et al.^73^	Retrospective cohort study	USA; National Cancer Institute SEER database	Men and women with primary diagnoses of oropharynx cancers	1975−2006 69.1 months (median)	8440	Cervix; 7 Oropharynx; 38	2.80 (1.28−5.32) 40.16 (28.42−55.12)	Total cohort has all H&N primary cancers (*N* = 75,087), Number of specificallyoropharynx primaries are a subset of the total and not reported separately in this article but assumed the same number as reported in ref. ^48^
Neumann et al.^58^	Retrospective cohort study	France; K2 database	Men and women with potentially HPV-related primary cancer diagnoses	1989−2004; 3.1 years (median)	6049 women 4078 men	Oropharyngeal; 3 Anal; 1 Oropharyngeal; 45	56.26 (11.31−164.38) 4.49 (0.06−24.97) 26.65 (19.44–35.66)	Incidence of second primary tongue and tonsil reported separately and pooled for this analysis
Sikora et al.^67^	Retrospective cohort study	USA; National Cancer Institute SEER database	Men with primary cancer diagnoses in the tongue or tonsil;	1973−2004 4.0 years median 1973−2004 4.3 years median	5912 10,752	*Tonsil primary*: Anal; 2 *Tongue primary*: Anal; 3 Penile; 1	3.1 (0.4–11.1) 2.3 (0.5−6.7) 0.9 (0.0–4.7)	Reported separately for primary tonsil and tongue cancers
Soderholm et al.^74^	Retrospective cohort study	Finland; Finnish Cancer Registry	Patients with primary diagnoses of cancer in the lip or oropharynx	1953−1989 Average follow-up not reported (minimum = 6 months)	3459	Oropharyngeal; 11	5.8 (2.8−10.0)	

### Anal cancer after a primary HPV-associated cancer or preinvasive tumour

**Fig. 2:**
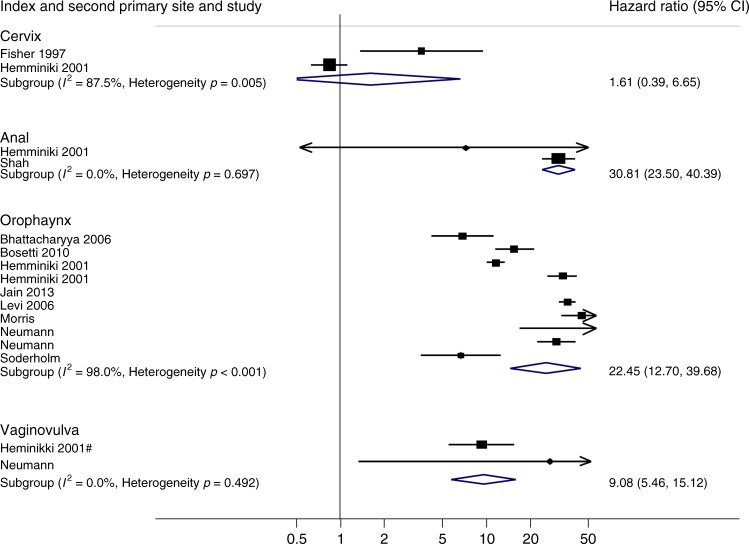
Standardised incidence ratios of second primary cancer after HPV-associated primary tumours at the same location

Two studies (two comparisons)^51,66^ reported incidence of second primary anal cancer following the same index cancer. Figure 2 shows that the combined SIR for these two studies was 30.81 (95% CI 23.5−40.39) and no evidence of heterogeneity or inconsistency between the studies (*p* = 0.697, *I*^2^ = 0).

**Fig. 3:**
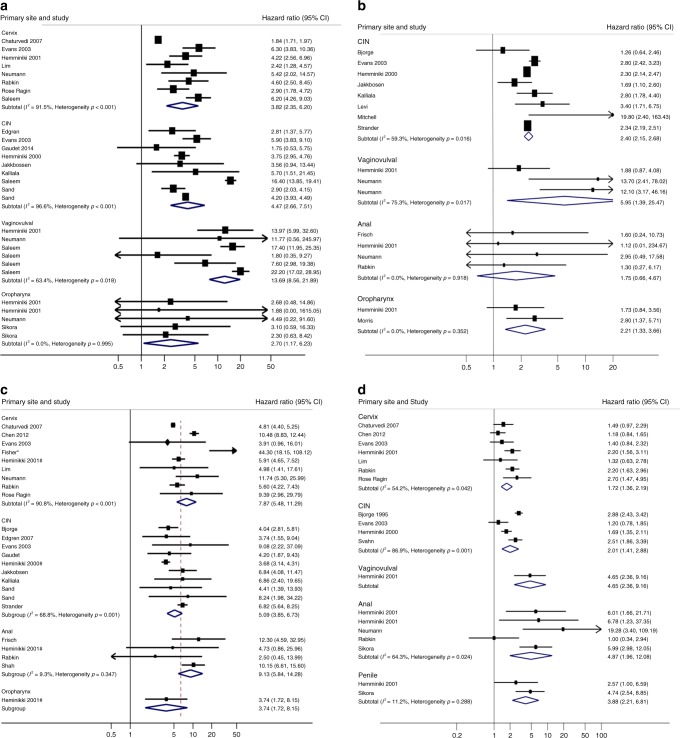
**a** Standardised incidence ratios of anal cancer after HPV-associated primary tumours. **b** Standardised incidence ratios of cervical cancer after HPV-associated primary tumours. **c** Standardised incidence ratios of vulvo-vaginal cancer after HPV-associated primary tumours. **d** Standardised incidence ratios of oropharyngeal cancer after HPV-associated primary tumours

Fourteen studies (28 comparisons)^44,47,48,51,54,58,62^ reported rates of second primary anal cancer after an initial diagnosis of an independent index HPV-associated cancer. While there is considerable heterogeneity between studies grouped by primary cervical (heterogeneity *p* < 0.001; *I*^2^ 91.5%), CIN (heterogeneity *p* < 0.001; *I*^2^ 96.36%) and vulvo-vaginal (heterogeneity *p* = 0.018; *I*^2^ 63.4%) cancers, and also evidence of significant variation between groups (test for interaction *p* < 0.001), the tendency towards an increase in risk is observed for all studies, and across each of the index sites. SIRs for individual index tumours ranged from 2.70 (95% CI 1.17−6.23) following an oropharyngeal index tumour to 13.69 (95% CI 8.56−21.89) after vulvo-vaginal index tumours (Fig. 3a and Table 1).

### Cervical cancer after a primary HPV-associated cancer or preinvasive tumour

Two studies (two comparisons)^49,51^ reported incidence of second primary cervical cancer following a primary cervix cancer. Figure 2 shows that the combined SIR for these two studies was 1.61 (95% CI 0.39−6.65) although there is evidence of heterogeneity and inconsistency between the studies (*p* = 0.005, *I*^2^ = 87.5%). Thirteen studies (17 comparisons)^43,48,51–53,55,57,59,63,65,73^ reported second primary cervical cancers after an independent primary HPV-associated cancer. While there is considerable heterogeneity between studies grouped by CIN (heterogeneity *p* = 0.016; *I*^2^ 59.3%) and vulvo-vaginal (heterogeneity *p* = 0.017; *I*^2^ 75.3%) index cancers, there is no evidence of variation in risk between groups (test for interaction *p* = 0.514). SIRs ranged from 1.75 (95% CI 0.66−4.67) following primary anal cancer to 5.95 (95% CI 1.39−25.47) following vulvo-vaginal cancer (Fig. 3b and Table 1).

### Vulvo-vaginal cancer after a primary HPV-associated cancer or preinvasive tumour

Two studies (two comparisons)^51,57^ reported incidence of second primary vulvo-vaginal cancer following the same index HAC. Figure 2 shows that the combined SIR for these two studies was 9.08 (95% CI 5.46−15.12) with no evidence of heterogeneity between the studies (*p* = 0.492, *I*^2^ = 0). Nineteen studies with 24 comparisons^43,44,47,54,57,58,60,63,65,66^ reported second primary vulvo-vaginal cancer (Fig. 3c). There is considerable heterogeneity and inconsistency between studies grouped by cervical (heterogeneity *p* < 0.001; *I*^2^ 90.8%) and CIN (heterogeneity *p* = 0.001; *I*^2^ 68.8%) index cancers, and evidence of variation in risk between index cancer groups (test for interaction *p* = 0.001). However, an increase in risk is observed for all except three of the individual studies and to each of the index sites. The SIRs for individual index tumours ranged from 3.74 (95% CI 1.72−8.15) for oropharyngeal index tumours to 9.13 (95% CI 5.84−14.28) for index anal cancers (Fig. 3c and Table 1).

### Oropharyngeal cancer after a primary HPV-associated cancer or preinvasive tumour

Eight studies (ten comparisons)^51,52,58,68,69,71,74^ reported incidence of second primary oropharyngeal cancers following a cancer at the same location. Figure 2 shows that the combined SIR for these studies was 22.45 (95% CI 12.70−39.68) with substantial evidence of heterogeneity between the studies (*p* < 0.001, *I*^2^ = 98%). Twelve studies (19 comparisons)^43,44,47,48,51,52,56,57,60,64,67^ reported second primary oropharyngeal cancer. There is considerable heterogeneity and inconsistency between studies grouped by cervical (heterogeneity *p* = 0.042; *I*^2^ 54.2%) and CIN (heterogeneity *p* < 0.001; *I*^2^ 86.9%) and anal (heterogeneity *p* = 0.024; *I*^2^ 64.3%) index cancers, and evidence of variation in risk between index cancer groups (test for interaction *p* < 0.001). However, a tendency towards an increase in risk is observed for the majority of individual studies, and for each of the index sites. The SIR for individual index tumours ranged from 1.72 (95% CI 1.36−2.19) for cervical index tumours to 4.87 (95% CI 1.96−12.08) for anal index tumours (Fig. 3d and Table 1). As cancers of the tonsil or tongue base are specific oropharyngeal tumours strongly related to the presence of HPV, we carried out a sensitivity analysis in which we limited the meta-analysis to the six studies that specifically reported incidence rates of second primary cancers at these sites.^43,44,56,60,64,67^ While there is less power overall, the magnitude and direction of the risks were similar to those obtained for any second cancer of the oropharynx (Figure S1, Supplementary material).

### Penile cancer after a primary HPV-associated cancer

Just three studies^65–67^ representing only nine cases reported second primary penile cancer, with SIRs ranging from 0.9 (0.0−4.7) to 2.93 (0.07−16.33). Formal meta-analysis was deemed inappropriate.

## Discussion

We have demonstrated that for patients diagnosed with HPV-associated invasive or preinvasive tumours, the risk of a second HPV-associated cancer at most sites is approximately a fivefold increase as compared with unaffected individuals; although for subsequent cervical cancers, this increase in risk is somewhat less (around 2-fold). There appears to be a particularly strong link between anal and vulvo-vaginal cancers, where either diagnosis confers around a tenfold increased risk of a second cancer at the other site. There is also a high rate of second cancers observed at the same anatomical site (acknowledging that it is difficult to differentiate recurrences from true second primary cancers from registry data in this context). For individuals this increased risk is likely to arise as a combination of exposure to high-risk HPV subtypes (so mediated by sexual behaviour) and subsequent inter- and intra-site transmission of HPV within individuals, and potentially host susceptibility where it has been suggested that mediators of immune clearance of HPV might play a role.^9,10^

Ours is the first systematic review and meta-analysis to have estimated the risk of developing a second primary HPV-associated cancer encompassing all anogenital and oropharyngeal sites. We have included data from 32 studies representing patients from 14 countries and spanning 77 years. Anticipating heterogeneity between the studies, we planned our analyses accordingly, using a random effects model to complete the meta-analysis, grouping studies according to the index primary cancer site. We also excluded studies that did not use a contemporaneous control from the meta-analysis as we felt that studies reporting SIRs were more reliable in terms of methodological quality. Application of the SIRs produced by this approach to current incidence rates^75^ (Table 2) gives estimates that are an order of magnitude less than those seen in studies reporting an institutional cohort of index cancers (Table S1). However, as patients included in these institutional cohort studies are likely to have been selected, and potentially followed up more intensely after primary treatment, they are more likely to have diagnosed early lesions than would be expected through a cancer registry approach. Hence, although the registry data gives a potentially more conservative estimate of second cancer risk, it is also likely to be more reliable and representative.

**Table 2:** Pooled SIRs of second HAC after primary tumours, and basal incidence, Europe and North America

Primary cancer	Secondary cancer, pooled SIR (95% CI)			
	Cervix	Vulvo-vaginal	Anal	Oropharyngeal
Cervix	1.61 (0.39−6.65)	7.76 (5.50−10.95)	3.82 (2.35−6.20)	1.72 (1.36−2.19)
CIN	2.40 (2.15−2.68)	5.09 (3.85−6.73)	4.47 (2.66−7.51)	2.01 (1.41−2.88)
Vulvo-vaginal	5.95 (1.39−25.47)	9.08 (5.46−15.12)	13.69 (8.56−21.89)	4.65 (2.36−9.16)
Anal	1.75 (0.66−4.67)	9.13 (5.84−14.28)	30.81 (23.50−40.39)	4.87 (1.96−6.81)
Penile	NA	NA	—	3.88 (2.21−6.81)
Oropharyngeal	2.21 (1.33−3.66)	3.74 (1.72−8.15)	2.70 (1.17−6.23)	22.45 (12.70−39.68)
Incidence (UK)	10 per 100,000	4.1 per 100,000	2 per 100,000	3−5 per 100,000
Incidence (Europe)^75^	15.2 per 100,000	0.8−4.1 per 100,000	1.2 per 100,000	7.9 per 100,000
Incidence (North America)^75^	8.1 per 100,000	2.5 per 100,000	1.8 per 100,000	6.1 per 100,000

Misclassification of tumours in registry-based studies may introduce over- or underestimation of second cancer incidence rates. For example, differentiating between true second cancers and local recurrences (and how this pertains to progression of preinvasive disease) in practice can be difficult, and lead to classification of local recurrences as second primary lesions. Some of the included studies reported attempts to account for this, notably by excluding second cancers at the same anatomical site that were identified within the first year after diagnosis (Table 1). In our meta-analyses, we have presented the rates of subsequent disease at the same site separately to try and avoid any overestimate of risk due to inclusion of local recurrences. Another opportunity for misclassification may arise due to the close anatomical proximity of anogenital cancers. Registry data might record a local recurrence that invades an adjacent organ as a second primary cancer. However, results from institutional series (that might be expected to suffer less from these problems—supplementary material Table S1) report higher rates of second cancers suggesting this issue has not significantly inflated the SIRs seen from the registries. Finally, difficulties in discriminating tumours arising in discrete sites within the oropharynx may have led to some misclassifications and as we cannot be completely confident of tumour classifications reported within the registry studies, there may be some over- or underestimation of risk that may have occurred in the individual studies. However, the results of our planned analysis based on risk of oropharynx cancers as reported, and our sensitivity analysis looking at risk of only tongue base or tonsil cancers are broadly in keeping with one another, thus suggesting our interpretation is robust to this.

Although we anticipated that heterogeneity might be an issue and attempted to address it in our preplanned analyses, by grouping studies according to initial and second primary cancers, statistical heterogeneity is still substantial. This is likely to be due to epidemiological differences between the studies, for example different extents of follow-up times, the range of time periods covered by the studies, changing demographics of cancers over time, different selection criteria for patients and differences in treatment regimes. Moderate to high heterogeneity has also been observed in other meta-analyses of second cancer data across a range of settings,^76^ with similar reasoning. In addition, as discussed above, over-or underestimation of second primary cancers due to difficulties in accurate classification within registry studies may also inflate the heterogeneity observed between the study results. However, almost all studies irrespective of the type and location of tumours show increases in risk of second cancer following initial primary cancer. The direction of the effect is broadly consistent, with the vast majority of studies indicating increased level of risk. The heterogeneity observed in these meta-analyses arises largely therefore due to differences in the magnitude of risk observed between studies. Therefore, while we cannot be certain of the true size of the risk, our results are indicative of an increase in risk for all of the sites assessed.

Another potential limitation is that data from studies with cervix as the primary site (whether preinvasive or invasive) predominate, given their relative incidence. It does mean that the majority of data included in our analyses are from female patients. Conversely, due to sparsity of available data, we have not been able to draw firm conclusions about the risk of second penile cancers, beyond the observation that an increased risk is consistent with the other sites of second HPV-associated tumours.

The registry data that underpins our meta-analysis were predominately derived from countries with cervical screening programmes. Effective screening routinely identifies individuals with precancerous conditions and thus reduces the subsequent risk of invasive disease. This may at least in part explain the smaller increase in risk seen for secondary cervix cancers compared to other sites. Equally hysterectomy might form part of the treatment of the initial HPV-associated cancer and as such further contribute to the lower risk of subsequent cervical cancer seen. Importantly though, cervical cancer and other HPV-associated cancers are particularly common in low and middle-income countries where screening programmes are not well established. Indeed, there is currently no coordinated surveillance after a diagnosis of a HPV-associated noncervical cancer for any population group, raising the concern that early diagnosis of curable cancer may be missed. Conversely, screening programmes (through over diagnosis) will expose patients to a range of detrimental side effects, for example in the treatment of AIN where a number of approaches are possible,^77^ and require prospective evaluation. This is currently being undertaken in the context of men who have sex with men (MSM) and anal cancer in the SPANC trial (study for the prevention of anal cancer).^78^ It should be noted that none of the studies included in the meta-analyses contained data on behavioural risk factors such as sexual behaviour, MSM etc. though it is likely that this will further modulate risk.

Based on our results, the diagnosis and treatment of index cancers presents an opportunity for secondary prevention, even when primary vaccination or screening is lacking. There could be the potential for therapeutic intervention using novel approaches in these patients to clear latent HPV infection or eradicate transformed cells. There is no evidence that the current prophylactic vaccines can eliminate transformed cells, though some data show that vaccination of subjects treated for HPV-associated precancers reduces the risk of new lesions in the genital tract. A small, nonrandomised cohort study of 202 patients with high-grade AIN^79^ showed fewer subsequent diagnoses at 2 years (HR 0.50; 95% CI, 0.26–0.98; *p* = 0.05) following quadrivalent HPV vaccination. A separate study^80^ of 737 patients with CIN2/3, treated with LEEP, also showed reduced rates of subsequent lesions in patients who subsequently received the quadrivalent HPV vaccine compared with a nonvaccinated group. Furthermore, retrospective analysis of data from randomised controlled trials of the HPV vaccine suggest that patients who developed a cervical lesion despite vaccination, and so were likely to have been infected with HPV prior to vaccination, were still relatively protected from subsequent recurrent/secondary HPV disease.^81^ There is also considerable interest in the development of therapeutic vaccines that stimulate an immune response against established infection. Pilot studies of such approaches suggest efficacy in CIN^82^ and larger trials including as adjuvant therapy after curative treatment of invasive cancers are in development. Finally, the growing field of immuno-oncology offers a number of approaches (for example immune checkpoint inhibitors) that might be utilised to eradicate HPV transformed cells, whether in reducing local recurrences or the development of second cancers.

In summary, there is a consistently raised incidence of each of the HPV-associated tumours as a second cancer after any such primary. Diagnosis and treatment of these index cancers presents a unique opportunity for the prevention of subsequent primary cancers. These data should inform patients and carers alike with respect to survivorship programmes. They also support new studies aimed at reducing the risks, whether through targeted screening of affected individuals, or trials of therapeutic approaches.

## Electronic supplementary material

Supplementary Materials

## References

[1] Plummer M (2016). Global burden of cancers attributable to infections in 2012: a synthetic analysis. Lancet Glob. Health.

[2] De Vuyst H, Clifford GM, Nascimento MC, Madeleine MM, Franceschi S (2009). Prevalence and type distribution of human papillomavirus in carcinoma and intraepithelial neoplasia of the vulva, vagina and anus: a meta-analysis. Int. J. Cancer.

[3] Koncar RF, Feldman R, Bahassi EM, Hashemi Sadraei N (2017). Comparative molecular profiling of HPV-induced squamous cell carcinomas. Cancer Med..

[4] Bansal N (2009). Primary therapy for early-stage cervical cancer: radical hysterectomy vs radiation. Am. J. Obstet. Gynecol..

[5] Goldstone SE, Johnstone AA, Moshier EL (2014). Long-term outcome of ablation of anal high-grade squamous intraepithelial lesions: recurrence and incidence of cancer. Dis. Colon Rectum.

[6] Ang KK (2014). Randomized phase III trial of concurrent accelerated radiation plus cisplatin with or without cetuximab for stage III to IV head and neck carcinoma: RTOG 0522. J. Clin. Oncol..

[7] James RD (2013). Mitomycin or cisplatin chemoradiation with or without maintenance chemotherapy for treatment of squamous-cell carcinoma of the anus (ACT II): a randomised, phase 3, open-label, 2 x 2 factorial trial. Lancet Oncol..

[8] Moscicki AB (2010). The role of sexual behavior and human papillomavirus persistence in predicting repeated infections with new human papillomavirus types. Cancer Epidemiol. Biomark. Prev..

[9] Levovitz C (2014). TGFbeta receptor 1: an immune susceptibility gene in HPV-associated cancer. Cancer Res..

[10] Chen D (2013). Genome-wide association study of susceptibility loci for cervical cancer. J. Natl. Cancer Inst..

[11] Schoenberg BS, Myers MH (1977). Statistical methods for studying multiple primary malignant neoplasms. Cancer.

[12] Wells, G. A., et al. The Newcastle-Ottawa Scale (NOS) for assessing the quality of nonrandomised studies in meta-analyses. Accessed 2017. http://www.ohri.ca/programs/clinical_epidemiology/oxford.htm

[13] Stroup DF (2000). Meta-analysis of observational studies in epidemiology: a proposal for reporting. Meta-analysis Of Observational Studies in Epidemiology (MOOSE) group. JAMA.

[14] Higgins JP, Thompson SG, Deeks JJ, Altman DG (2003). Measuring inconsistency in meta-analyses. BMJ.

[15] DerSimonian R, Kacker R (2007). Random-effects model for meta-analysis of clinical trials: an update. Contemp. Clin. Trials.

[16] Fisher DJ (2015). Two-stage individual participant data meta-analysis and generalised forest plots. STATA J..

[17] Robertson JH, Woodend BE, Crozier EH, Patterson A (1987). Risk of recurrence after treatment of severe intraepithelial neoplasia of the cervix. A follow-up of 896 patients. Ulst. Med. J..

[18] Soutter WP, Sasieni P, Panoskaltsis T (2006). Long-term risk of invasive cervical cancer after treatment of squamous cervical intraepithelial neoplasia. Int. J. Cancer.

[19] Pearson SE, Whittaker J, Ireland D, Monaghan JM (1989). Invasive cancer of the cervix after laser treatment. Br. J. Obstet. Gynaecol..

[20] McIndoe WA, McLean MR, Jones RW, Mullins PR (1984). The invasive potential of carcinoma in situ of the cervix. Obstet. Gynecol..

[21] Kolstad P, Klem V (1976). Long-term follow up of 1121 cases of carcinoma in situ. Obstet. Gynecol..

[22] Andersch B, Moinian M (1982). Diagnostic and therapeutic viewpoints on cervical intraepithelial neoplasia. 10-Year follow-up of a conization material. Gynecol. Obstet. Invest..

[23] Li Z, Barron S, Hong W, Karunamurthy A, Zhao C (2013). Surveillance for recurrent cancers and vaginal epithelial lesions in patients with invasive cervical cancer after hysterectomy: are vaginal cytology and high-risk human papillomavirus testing useful?. Am. J. Clin. Path..

[24] Liu L (2011). Prevalence of multiple malignancies in the Netherlands in 2007. Int. J. Cancer.

[25] van de Nieuwenhof HP (2009). Vulvar squamous cell carcinoma development after diagnosis of VIN increases with age. Eur. J. Cancer.

[26] Mitchell MF (1993). Second genital primary squamous neoplasms in vulvar carcinoma: viral and histopathologic correlates. Obstet. Gynecol..

[27] Tiwana MS (2014). Incidence of second metachronous head and neck cancers: population-based outcomes over 25 years. Laryngoscope.

[28] van der Haring IS, Schaapveld MS, Roodenburg JL, de Bock GH (2009). Second primary tumours after a squamous cell carcinoma of the oral cavity or oropharynx using the cumulative incidence method. Int. J. Oral Maxillofac. Surg..

[29] Gan SJ (2013). Incidence and pattern of second primary malignancies in patients with index oropharyngeal cancers versus index nonoropharyngeal head and neck cancers. Cancer.

[30] Kramer FJ, Janssen M, Eckardt A (2004). Second primary tumours in oropharyngeal squamous cell carcinoma. Clin. Oral Invest..

[31] Leon X (1999). Second neoplasm in patients with head and neck cancer. Head Neck.

[32] Hsu YB (2008). Second primary malignancies in squamous cell carcinomas of the tongue and larynx: an analysis of incidence, pattern, and outcome. J. Chin. Med. Assoc..

[33] Crocetti E, Barchielli A (1998). Risk of metachronous primary cancers in women with cervical tumor—an Italian population-based study. Gynecol. Oncol..

[34] Melnikow J, McGahan C, Sawaya GF, Ehlen T, Coldman A (2009). Cervical intraepithelial neoplasia outcomes after treatment: long-term follow-up from the British Columbia Cohort Study. J. Natl. Cancer Inst..

[35] Pettersson F, Malker B (1989). Invasive carcinoma of the uterine cervix following diagnosis and treatment of in situ carcinoma. Record linkage study within a National Cancer Registry. Radiother. Oncol..

[36] Arnold M (2014). Second primary cancers in survivors of cervical cancer in The Netherlands: implications for prevention and surveillance. Radiother. Oncol..

[37] Balamurugan A (2008). Potential role of human papillomavirus in the development of subsequent primary in situ and invasive cancers among cervical cancer survivors. Cancer.

[38] Jegu J (2014). The effect of patient characteristics on second primary cancer risk in France. Bmc Cancer.

[39] Newell GR, Krementz ET, Roberts JD (1975). Excess occurrence of cancer of the oral cavity, lung, and bladder following cancer of the cervix. Cancer.

[40] Sturgeon SR, Curtis RE, Johnson K, Ries L, Brinton LA (1996). Second primary cancers after vulvar and vaginal cancers. Am. J. Obstet. Gynecol..

[41] Kapp DS, Fischer D, Grady KJ, Schwartz PE (1982). Subsequent malignancies associated with carcinoma of the uterine cervix: including an analysis of the effect of patient and treatment parameters on incidence and sites of metachronous malignancies. Int. J. Radiat. Oncol. Biol. Phys..

[42] Lee JY, Perez CA, Ettinger N, Fineberg BB (1982). The risk of second primaries subsequent to irradiation for cervix cancer. Int. J. Radiat. Oncol. Biol. Phys..

[43] Bjorge T, Hennig EM, Skare GB, Soreide O, Thoresen SO (1995). Second primary cancers in patients with carcinoma in situ of the uterine cervix. The Norwegian experience 1970–1992. Int. J. Cancer.

[44] Chaturvedi AK (2007). Second cancers among 104,760 survivors of cervical cancer: evaluation of long-term risk. J. Natl. Cancer Inst..

[45] Chaturvedi AK (2009). Second cancers after squamous cell carcinoma and adenocarcinoma of the cervix. J. Clin. Oncol..

[46] Chen CY (2012). Risk of second primary malignancies in women with cervical cancer: a population-based study in Taiwan over a 30-year period. Gynecol. Oncol..

[47] Edgren G, Sparen P (2007). Risk of anogenital cancer after diagnosis of cervical intraepithelial neoplasia: a prospective population-based study. Lancet Oncol..

[48] Evans HS, Newnham A, Hodgson SV, Moller H (2003). Second primary cancers after cervical intraepithelial neoplasia III and invasive cervical cancer in Southeast England. Gynecol. Oncol..

[49] Fisher G, Harlow SD, Schottenfeld D (1997). Cumulative risk of second primary cancers in women with index primary cancers of uterine cervix and incidence of lower anogenital tract cancers, Michigan, 1985–1992. Gynecol. Oncol..

[50] Gaudet M, Hamm J, Aquino-Parsons C (2014). Incidence of ano-genital and head and neck malignancies in women with a previous diagnosis of cervical intraepithelial neoplasia. Gynecol. Oncol..

[51] Hemminki K, Jiang Y, Dong C (2001). Second primary cancers after anogenital, skin, oral, esophageal and rectal cancers: etiological links?. Int. J. Cancer.

[52] Hemminki K, Dong C, Vaittinen P (2000). Second primary cancer after in situ and invasive cervical cancer. Epidemiology.

[53] Jakobsson M, Pukkala E, Paavonen J, Tapper AM, Gissler M (2011). Cancer incidence among Finnish women with surgical treatment for cervical intraepithelial neoplasia, 1987−2006. Int. J. Cancer.

[54] Kalliala I, Anttila A, Pukkala E, Nieminen P (2005). Risk of cervical and other cancers after treatment of cervical intraepithelial neoplasia: retrospective cohort study. BMJ.

[55] Levi F, Randimbison L, La Vecchia C, Franceschi S (1996). Incidence of invasive cancers following carcinoma in situ of the cervix. Br. J. Cancer.

[56] Lim MC (2016). Second primary cancer after diagnosis and treatment of cervical cancer. Cancer Res. Treat..

[57] Mitchell H, Medley G, Carlin JB (1990). Risk of subsequent cytological abnormality and cancer among women with a history of cervical intraepithelial neoplasia: a comparative study. Cancer Causes Control.

[58] Neumann F (2016). Risk of second primary cancer after a first potentially-human papillomavirus-related cancer: a population-based study. Prev. Med..

[59] Rabkin CS, Biggar RJ, Melbye M, Curtis RE (1992). Second primary cancers following anal and cervical carcinoma: evidence of shared etiologic factors. Am. J. Epidemiol..

[60] Rose Ragin CC, Taioli E (2008). Second primary head and neck tumor risk in patients with cervical cancer—SEER data analysis. Head Neck.

[61] Saleem AM (2011). Risk of anal cancer in a cohort with human papillomavirus-related gynecologic neoplasm. Obstet. Gynecol..

[62] Sand FL (2016). Long-term risk for noncervical anogenital cancer in women with previously diagnosed high-grade cervical intraepithelial neoplasia: a Danish Nationwide Cohort Study. Cancer Epidemiol. Biomark. Prev..

[63] Strander B, Andersson-Ellstrom A, Milsom I, Sparen P (2007). Long term risk of invasive cancer after treatment for cervical intraepithelial neoplasia grade 3: population based cohort study. BMJ.

[64] Svahn MF (2016). Risk of head-and-neck cancer following a diagnosis of severe cervical intraepithelial neoplasia: a nationwide population-based cohort study in Denmark. Gynecol. Oncol..

[65] Frisch M, Olsen JH, Melbye M (1994). Malignancies that occur before and after anal cancer: clues to their etiology. Am. J. Epidemiol..

[66] Shah BK, Budhathoki N (2015). Second primary malignancy in anal carcinoma—a US population-based study. Anticancer Res..

[67] Sikora AG, Morris LG, Sturgis EM (2009). Bidirectional association of anogenital and oral cavity/pharyngeal carcinomas in men. Arch. Otolaryngol. Head Neck Surg..

[68] Bhattacharyya N (2006). An assessment of risk factors for the development of a second primary malignancy in the head and neck. Ear Nose Throat J..

[69] Bosetti C (2011). High constant incidence rates of second primary cancers of the head and neck: a pooled analysis of 13 cancer registries. Int. J. Cancer.

[70] Chuang SC (2008). Risk of second primary cancer among patients with head and neck cancers: a pooled analysis of 13 cancer registries. Int. J. Cancer.

[71] Jain KS, Sikora AG, Baxi SS, Morris LG (2013). Synchronous cancers in patients with head and neck cancer: risks in the era of human papillomavirus-associated oropharyngeal cancer. Cancer.

[72] Levi F, Te VC, Randimbison L, Maspoli M, La Vecchia C (2006). Second primary oral and pharyngeal cancers in subjects diagnosed with oral and pharyngeal cancer. Int. J. Cancer.

[73] Morris LG, Sikora AG, Patel SG, Hayes RB, Ganly I (2011). Second primary cancers after an index head and neck cancer: subsite-specific trends in the era of human papillomavirus-associated oropharyngeal cancer. J. Clin. Oncol..

[74] Soderholm AL, Pukkala E, Lindqvist C, Teppo L (1994). Risk of new primary cancer in patients with oropharyngeal cancer. Br. J. Cancer.

[75] de Martel C, Plummer M, Vignat J, Franceschi S (2017). Worldwide burden of cancer attributable to HPV by site, country and HPV type. Int. J. Cancer.

[76] Grantzau T, Overgaard J (2016). Risk of second non-breast cancer among patients treated with and without postoperative radiotherapy for primary breast cancer: a systematic review and meta-analysis of population-based studies including 522,739 patients. Radiother. Oncol..

[77] Pernot S (2018). Comparison of anal cancer screening strategies including standard anoscopy, anal cytology, and HPV genotyping in HIV-positive men who have sex with men. Br. J. Cancer.

[78] Machalek DA (2013). The Study of the Prevention of Anal Cancer (SPANC): design and methods of a three-year prospective cohort study. Bmc Public Health.

[79] Swedish KA, Factor SH, Goldstone SE (2012). Prevention of recurrent high-grade anal neoplasia with quadrivalent human papillomavirus vaccination of men who have sex with men: a nonconcurrent cohort study. Clin. Infect. Dis..

[80] Kang WD, Choi HS, Kim SM (2013). Is vaccination with quadrivalent HPV vaccine after loop electrosurgical excision procedure effective in preventing recurrence in patients with high-grade cervical intraepithelial neoplasia (CIN2-3)?. Gynecol. Oncol..

[81] Joura EA (2012). Effect of the human papillomavirus (HPV) quadrivalent vaccine in a subgroup of women with cervical and vulvar disease: retrospective pooled analysis of trial data. BMJ.

[82] Trimble CL (2015). Safety, efficacy, and immunogenicity of VGX-3100, a therapeutic synthetic DNA vaccine targeting human papillomavirus 16 and 18 E6 and E7 proteins for cervical intraepithelial neoplasia 2/3: a randomised, double-blind, placebo-controlled phase 2b trial. Lancet.

