# Supplementary material for: Increased risk of second cancers at sites associated with HPV after a prior HPV-associated malignancy, a systematic review and meta-analysis
Source: Br J Cancer. 2018 Nov 28;120(2):256–68. doi: 10.1038/s41416-018-0273-9 (PMC6342987; doi:10.1038/s41416-018-0273-9)
Supplement: Supplementary file 1 — Supplementary Materials [file 41416_2018_273_MOESM1_ESM.docx]

**Supplementary Materials;**

Table S1 – Second HPV associated cancers in cohort studies (absolute numbers and % cases)

Table S2 – Second HPV associated cancers in registries where CI not reported – absolute numbers and reported SIR

Figure S1 - Standardised incidence ratios of specific tonsil or tongue base cancer after HPV associated primary tumours

Appendix 1 – Search Strategy

Appendix 2 – MOOSE checklist

**Supplementary Materials;**

**Table S1 – Second HPV associated cancers in cohort studies (absolute numbers and % cases) *=**CIN

| **Author** | **Primary Cancer** | **N** | **Second Cancer, N (%)** | | | | | | |
| --- | --- | --- | --- | --- | --- | --- | --- | --- | --- |
|  |  |  | **Cervix** | **Vulval** | **Vaginal** | **Anal** | **Penile** | **Oropharynx** | **HNSCC** |
| Robertson et al 1987^17^ | CIN | 896 | 5 (0.56) |  | 7 (0.78) |  |  |  |  |
| Soutter et al 1997 ^18^ | CIN | 2116 | 33 (1.56) |  |  |  |  |  |  |
| Pearson et al 1989 ^19^ | CIN | 4222 | 9 (0.21) |  |  |  |  |  |  |
| McIndoe et al 1984^20^ | CIN | 948 | 12 (1.23) |  |  |  |  |  |  |
| Kolstad et al 1976 ^21^ | CIN | 1121 | 12 (1.07) |  |  |  |  |  |  |
| Andersch et al 1982^22^ | CIN | 429 | 2 (0.47) |  |  |  |  |  |  |
| Li et al 2013 ^23^ | Cervix | 147 |  | 22 (15) |  |  |  |  |  |
| Liu et al 2011 ^24^ | Female Genital | 28254 | 418 anogenital (1.48) | | | |  | 22 (0.08) |  |
| Neiwenhot et al 2009 ^25^ | VIN | 1826 | 471*(25.79) | 104 (5.7) |  | 89 (4.9) |  |  |  |
| Mitchell et al 1993 ^26^ | Vulval | 169 | 17 (10.06) |  | 2 (1.2) |  |  |  |  |
| Liu et al 2011 ^24^ | Male Genital | 8446 | n/a | n/a | n/a |  | 6 (0.07) | 2 (0.02) |  |
| Tiwana et al 2014^27^ | Oropharynx | 348 |  |  |  |  |  | 27 (7.76) |  |
| Van der Haring et al 2009 ^28^ | Oropharynx | 917 |  |  |  |  |  | 65 (7.09) |  |
| Gan et al 2013 ^29^ | Oropharynx | 85 |  |  |  |  |  | 2 (2.35) |  |
| Kramer et al 2004 ^30^ | Oropharynx | 981 |  |  |  |  |  | 23 (2.34) | 90 (9.17) |
| Leon et al 1999 ^31^ | Oropharynx | 369 |  |  |  |  |  |  | 36 (9.76) |
| Liu et al 2011 ^24^ | Oropharynx | 15227 | 18 anogenital (0.12) | | | | 18 (0.12) | 423 (2.78) |  |
| Hsu et al 2008 ^32^ | Tongue | 146 |  |  |  |  |  |  | 13 (8.9) |

**Table S2 – Second HPV associated cancers in registries where CI not reported – absolute numbers and reported SIR.** *=person years at risk, # described as white and black female patients respectively.

| **Author** | **Primary Cancer** | **N** | **Second Cancer (SIR)** | | | | | | | |
| --- | --- | --- | --- | --- | --- | --- | --- | --- | --- | --- |
|  |  |  | **Cervix** | **Vulval** | **Vaginal** | **Anal** | **Penile** | **Oropharynx** | **HNSCC** | **Any HPV** |
| Crocetti+Barchielli 1998 ^33^ | CIN | 990 | 1 (2.72) |  |  |  |  | 1 (25.0) |  |  |
| Melnikow et al 2009 ^34^ | CIN | 37142 | 145 (6.17) |  |  |  |  |  |  |  |
| Pettersson et al 1989 ^35^ | CIN | 56117 | 211 (2.40) |  |  |  |  |  |  |  |
| Arnold et al 2014 ^36^ | Cervix | 12048 |  |  |  |  |  |  |  | 32 (2.0) |
| Balamurugan et al 2008 ^37^ | Cervix | 23409 | 20 (1.38) | 18 (5.74) | 33 (29.87) |  |  | 16 (1.68) |  |  |
| Jegu et al 2014 ^38^ | Cervix | 28112* |  | 10 (7.82) | |  |  |  |  |  |
|  | Vulvo-vaginal | 4219* | 6 (8.87) |  |  |  |  |  | 3 (6.37) |  |
| Newell et al 1975 ^39^ | Cervix# | 2185 and 2333* |  |  |  | 3 (2.0) and 4 (1.5) |  | 3 (5.3) and 3 (3.8) |  |  |
| Sturgeon et al 1996 ^40^ | Vaginal in situ | 461 | 1 (1.8) | 0 |  | 0 |  |  | 0 |  |
|  | VIN | 2898 | 5 (1.8) |  | 1 (4.4) | 6 (17.3) |  |  | 12 (5.0) |  |
|  | Vagina | 888 | 2 (2.7) | 1 (3.8) |  | 1 (7.8) |  |  |  |  |
|  | Vulva | 2685 | 3 (1.1) |  | 2 (4.8) | 3 (6.0) |  |  | 5 (1.6) |  |
|  | Cervix | 16,110 |  | 18 (5.2) | 19 (13.6) | 9 (4.7) |  |  | 26 (2.0) |  |
| Kapp et al 1982^41^ | Cervix | 763 |  | 5 (6.58) | |  |  | 1 (1.4) |  |  |
| Lee et al 1982 ^42^ | Cervix | 1048 |  |  | 2 (0.82) |  |  |  |  |  |

**Figure S1 - Standardised incidence ratios of specific tonsil or tongue base cancer after HPV associated primary tumours**


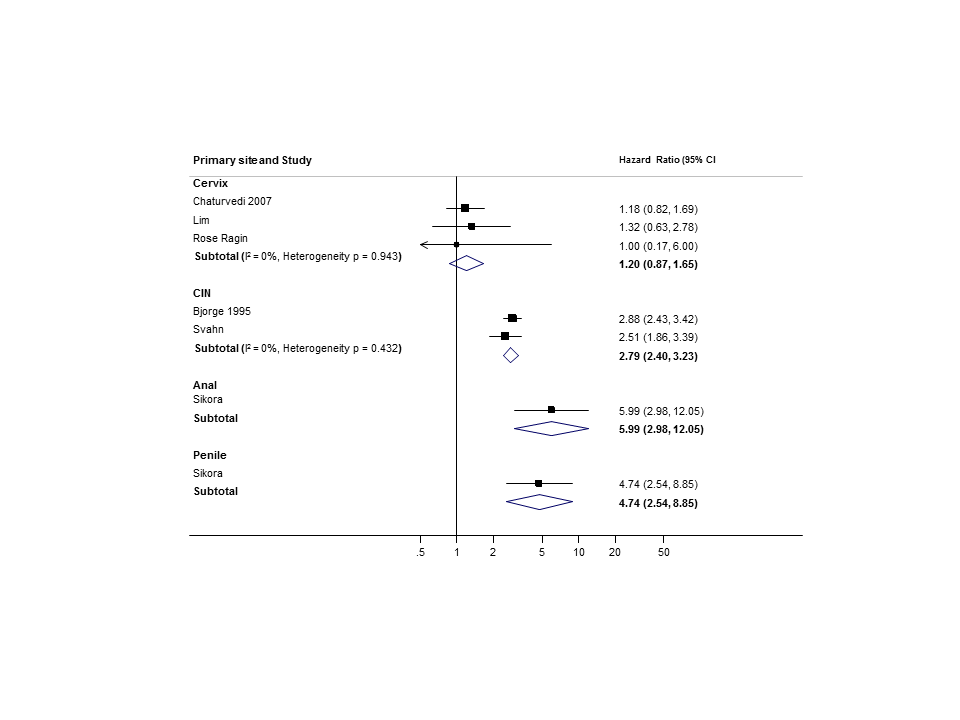


**Appendix 1 – Search Strategy;**

**HPV_final search strategy (saved March 5th 2015)**

| 1. exp Papillomavirus Infections/ |  |
| --- | --- |
| 2. exp Papillomaviridae/ |  |
| 3. exp HPV-11/ |  |
| 4. exp HPV-6/ |  |
| 5. exp HPV-16/ |  |
| 6. exp HPV-18/ |  |
| 7. Papillomavir$.mp. |  |
| 8. Papillomavir$.tw,ot. |  |
| 9. (Papilloma adj virus).tw,ot. |  |
| 10. (Papillomavirus adj2 human).mp. |  |
| 11. HPV.mp. |  |
| 12. HPV. tw,ot. |  |
| 13. (Cancer adj2 HPV).mp. |  |
| 14. (Cancer adj2 human papillomavirus).mp. |  |
| 15. Malignancy adj2 HPV |  |
| 16. Malignancy adj2 human papillomavirus |  |
| 17. or/1-16 |  |
| 18. exp Uterine Cervical Neoplasms/ |  |
| 19. Cancer of Cervix.tw,ot. |  |
| 20. Cancer of the Cervix.tw,ot. |  |
| 21. Cancer of the Uterine Cervix.tw,ot. |  |
| 22. Cervical Cancer.tw,ot. |  |
| 23. Cervical Neoplasms.tw,ot. |  |
| 24. Cervix Cancer.tw,ot. |  |
| 25. Cervix Neoplasms.tw,ot. |  |
| 26. Neoplasms, Cervical.tw,ot. |  |
| 27. Neoplasms, Cervix.tw,ot. |  |
| 28. Uterine Cervical Cancer.tw,ot. |  |
| 29. (Cervi$ adj3 cancer).mp. |  |
| 30. (Cervi$ adj3 carcinoma).mp. |  |
| 31. exp Vaginal Neoplasms/ |  |
| 32. Cancer of the Vagina.tw,ot. |  |
| 33. Cancer of Vagina.tw,ot. |  |
| 34. Neoplasms, Vaginal.tw,ot. |  |
| 35. Vagina Cancer.tw,ot. |  |
| 36. Vagina Neoplasms.tw,ot. |  |
| 37. Vaginal Cancer.tw,ot. |  |
| 38. (vagin$ adj3 cancer).mp. |  |
| 39. (vagin$ adj3 carcinoma).mp. |  |
| 40. vagina$ intraepithelial neoplasia.mp. |  |
| 41. vaginal epithelial lesions.mp. |  |
| 42. VAIN.mp. |  |
| 43. exp Vulvar Neoplasms/ |  |
| 44. Cancer of the Vulva.tw,ot. |  |
| 45. Cancer of Vulva.tw,ot. |  |
| 46. Neoplasms, Vulvar.tw,ot. |  |
| 47. Vulva Cancer.tw,ot. |  |
| 48. Vulva Neoplasms.tw,ot. |  |
| 49. Vulvar Cancer.tw,ot. |  |
| 50. (vulv$ adj3 cancer).mp. |  |
| 51. (vulv$ adj3 carcinoma).mp. |  |
| 52. vulva$ intraepithelial neoplasia.mp. |  |
| 53. exp Anus Neoplasms/ |  |
| 54. Anal Cancer.tw,ot. |  |
| 55. Anal Neoplasms.tw,ot. |  |
| 56. Cancer of Anus.tw,ot. |  |
| 57. Cancer of the Anus.tw,ot. |  |
| 58. Neoplasms, Anal.tw,ot. |  |
| 59. Neoplasms, Anus.tw,ot. |  |
| 60. (Ano$genital adj2 cancer).mp. |  |
| 61. (anal adj3 cancer).mp. |  |
| 62. (anal adj3 carcinoma).mp. |  |
| 63. (anus adj3 cancer).mp. |  |
| 64. (anus adj3 carcinoma).mp. |  |
| 65. anal intraepithelial neoplasia.mp. |  |
| 66. AIN.mp. |  |
| 67. exp Oropharyngeal Neoplasms/ |  |
| 68. Cancer of Oropharnyx.tw,ot. |  |
| 69. Cancer of the Oropharynx.tw,ot. |  |
| 70. Neoplasms, Oropharyngeal.tw,ot. |  |
| 71. Oropharyngeal Cancer.tw,ot. |  |
| 72. Oropharynx Cancer.tw,ot. |  |
| 73. Oropharynx Neoplasms.tw,ot. |  |
| 74. exp Penile Neoplasms/ |  |
| 75. Cancer of Penis.tw,ot. |  |
| 76. Cancer of the Penis.tw,ot. |  |
| 77. Neoplasms, Penile.tw,ot. |  |
| 78. Neoplasms, Penis.tw,ot. |  |
| 79. Penile Cancer.tw,ot. |  |
| 80. Penis Cancer.tw,ot. |  |
| 81. Penis Neoplasms.tw,ot. |  |
| 82. (Peni$ adj3 cancer).mp. |  |
| 83. (Peni$ adj3 carcinoma).mp. |  |
| 84. Peni$ intraepithelial neoplasia.mp. |  |
| 85. PIN.mp. |  |
| 86. exp Cervical Intraepithelial Neoplasia/ |  |
| 87. Cervical Intraepithelial Neoplasia.tw,ot. |  |
| 88. Cervical Intraepithelial Neoplasms.tw,ot. |  |
| 89. Intraepithelial Neoplasia, Cervical.tw,ot. |  |
| 90. Neoplasia, Cervical Intraepithelial.tw,ot. |  |
| 91. CIN.tw,ot. |  |
| 92. exp Neoplasms, Second Primary/ |  |
| 93. Cancer, Second Primary.tw,ot. |  |
| 94. Metachronous Neoplasms.tw,ot. |  |
| 95. Metachronous Second Primary Neoplasms.tw,ot. |  |
| 96. Neoplasms, Metachronous.tw,ot. |  |
| 97. Neoplasms, Metachronous Second Primary.tw,ot. |  |
| 98. Second Cancer.tw,ot. |  |
| 99. Second Malignancy.tw,ot. |  |
| 100. Second Neoplasm.tw,ot. |  |
| 101. Second Primary Neoplasms.tw,ot. |  |
| 102. Second Primary Neoplasms, Metachronous.tw,ot. |  |
| 103. (Subsequent adj cancer).mp. |  |
| 104. (Incident adj primary cancer).mp. |  |
| 105. (Metachronous adj primary cancer).mp. |  |
| 106. exp Incidence/ |  |
| 107. incidence.tw,ot. |  |
| 108. Epidemiologic Studies/ |  |
| 109. exp Case-Control Studies/ |  |
| 110. exp Cohort Studies/ |  |
| 111. Cross-Sectional Studies/ |  |
| 112. (epidemiologic$ adj (study or studies)).tw,ot. |  |
| 113. case control.tw,ot. |  |
| 114. (cohort adj (study or studies)).tw,ot. |  |
| 115. cross sectional.tw,ot. |  |
| 116. cohort analy$.tw,ot. |  |
| 117. (follow up adj (study or studies)).tw,ot. |  |
| 118. longitudinal.tw,ot. |  |
| 119. retrospective$.tw,ot. |  |
| 120. prospective$.tw,ot. |  |
| 121. (observ$ adj3 (study or studies)).tw,ot. |  |
| 122. exp "Review Literature as topic"/ |  |
| 123. exp Technology Assessment, Biomedical/ |  |
| 124. exp Meta-analysis as topic/ |  |
| 125. Meta-analysis.pt. |  |
| 126. hta.tw,ot. |  |
| 127. (health technology adj6 assessment$).tw,ot. |  |
| 128. (meta analy$ or metaanaly$ or meta?analy$).tw,ot. |  |
| 129. ((review$ or search$) adj10 (literature$ or medical database$ or medline or pubmed or embase or cochrane or cinahl or psycinfo or psyclit or healthstar or biosis or current content$ or systemat$)).tw,ot. |  |
| 130. or/18-91 |  |
| 131. or/92-105 |  |
| 132. 106 or 107 |  |
| 133. or/108-129 |  |
| 134. 17 and 131 |  |
| 135. 130 and 131 |  |
| 136. 131 and 132 |  |
| 137. 17 and 130 and 132 |  |
| 138. 134 or 135 or 136 or 137 |  |
| 139. 133 and 138 |  |

**Appendix 2 – MOOSE checklist;**

| Item No | Recommendation | Reported on Page No |
| --- | --- | --- |
| Reporting of background should include | | |
| 1 | Problem definition | page 5, background para 2 |
| 2 | Hypothesis statement | page 5, background para 3 |
| 3 | Description of study outcome(s) | page 5, background para 3 |
| 4 | Type of exposure or intervention used | page 5, background para 3 |
| 5 | Type of study designs used | page 6, methods study eligibility |
| 6 | Study population | page 6, methods study eligibility |
| Reporting of search strategy should include | | |
| 7 | Qualifications of searchers (eg, librarians and investigators) | page 1 |
| 8 | Search strategy, including time period included in the synthesis and key words | page 6, study identification |
| 9 | Effort to include all available studies, including contact with authors | page 6, study identification |
| 10 | Databases and registries searched | page 6, study identification |
| 11 | Search software used, name and version, including special features used (eg, explosion) | page 6, study identification |
| 12 | Use of hand searching (eg, reference lists of obtained articles) | page 6, study identification |
| 13 | List of citations located and those excluded, including justification | page 17, figure 1 |
| 14 | Method of addressing articles published in languages other than English | page 6, study eligibility |
| 15 | Method of handling abstracts and unpublished studies | page 7, study identification |
| 16 | Description of any contact with authors | Not required |
| Reporting of methods should include | | |
| 17 | Description of relevance or appropriateness of studies assembled for assessing the hypothesis to be tested | page 6, methods para 2 |
| 18 | Rationale for the selection and coding of data (eg, sound clinical principles or convenience) | page 6, methods para 3 |
| 19 | Documentation of how data were classified and coded (eg, multiple raters, blinding and interrater reliability) | page 7, methods para 2 |
| 20 | Assessment of confounding (eg, comparability of cases and controls in studies where appropriate) | page 7, methods para 3 |
| 21 | Assessment of study quality, including blinding of quality assessors, stratification or regression on possible predictors of study results | page 7, methods para 3 |
| 22 | Assessment of heterogeneity | page 9-11 results |
| 23 | Description of statistical methods (eg, complete description of fixed or random effects models, justification of whether the chosen models account for predictors of study results, dose-response models, or cumulative meta-analysis) in sufficient detail to be replicated | page 8, methods para 3 |
| 24 | Provision of appropriate tables and graphics | pages 17 and 20, figure 3 and table 1 and S1,S2 |
| Reporting of results should include | | |
| 25 | Graphic summarizing individual study estimates and overall estimate | page17, figures 3 |
| 26 | Table giving descriptive information for each study included | page 20, table 1 |
| 27 | Results of sensitivity testing (eg, subgroup analysis) | page 9-11, results |
| 28 | Indication of statistical uncertainty of findings | page 11-12, discussion |
| Reporting of discussion should include | | |
| 29 | Quantitative assessment of bias (eg, publication bias) | page 12, discussion |
| 30 | Justification for exclusion (eg, exclusion of non-English language citations) | Not applicable |
| 31 | Assessment of quality of included studies | page 7, results and page 12, discussion |
| Reporting of conclusions should include | | |
| 32 | Consideration of alternative explanations for observed results | page 12-13, discussion |
| 33 | Generalization of the conclusions (ie, appropriate for the data presented and within the domain of the literature review) | page 13, discussion |
| 34 | Guidelines for future research | page 14, discussion |
| 35 | Disclosure of funding source | page 16, acknowledgements |
